# Microwave effects in the dilute acid hydrolysis of cellulose to 5-hydroxymethylfurfural

**DOI:** 10.1038/s41598-018-26107-y

**Published:** 2018-05-16

**Authors:** Nick Sweygers, Niels Alewaters, Raf Dewil, Lise Appels

**Affiliations:** KU Leuven, Department of Chemical Engineering, Process and Environmental Technology Lab, J. De Nayerlaan 5, B-2860 Sint-Katelijne-Waver, Belgium

## Abstract

In this study, the effect of microwaves on the production of 5-hydroxymethylfurfural (HMF) in a biphasic system was evaluated via a kinetic analysis. The reaction system consisted of an acidified aqueous phase and methyl isobutyl ketone (MIBK) as an organic phase, in which HMF is extracted directly upon formation during the reaction. Two identically shaped reactors were used to assess the influence of microwaves on the production of HMF. A borosilicate glass reactor was used to heat the reaction mixture via microwaves directly, whereas the silicon carbide (SiC) wall of the second reactor absorbed all microwaves and hence the reactor content was heated via convective heat transfer. An identical temperature profile was imposed on both reactors. Cellulose, glucose and fructose were chosen as feedstocks for the conversion to HMF. It was observed that microwaves have a significant effect on the reactions. The hydrolysis of cellulose to glucose was a 2.3 folds faster in the presence of microwaves at the process conditions (0.046 M HCl, 177 °C). The isomerization of glucose to fructose showed a similar increase (factor 2.5). The required energy input for the reaction was systematically higher for the SiC reactor.

## Introduction

5-hydroxymethylfurfural (HMF) is a key bio-based platform chemical for the production of a broad spectrum of fine chemicals. HMF is the precursor of 2,5-furandicarboxylic acid (FDCA), which is a potential substitute for the fossil based terephthalic acid. Terephthalic acid is the precursor of polyethylene terephthalate (PET), and hence the use of HMF has a huge potential in the bio-plastics market^1^. In recent years, research on the use of various lignocellulosic raw materials for the synthesis of HMF has been performed^2,3^. However, an efficient and reliable way of producing HMF remains a major bottleneck for industrial scale application in bio-refinery plants^4^. HMF can be produced through conversion of monomeric sugars (such as glucose or fructose), which is a fairly easy and efficient process with a high selectivity and yield^5–7^. However, the associated costs for using the monomeric sugars are high and their availability is lower compared to lignocellulosic biomass. On the other hand, the conversion of lignocellulosic biomass is difficult since it generally contains high product impurities such as lignin, waxes and lipids, which reduce the HMF yield^8^. Nevertheless, lignocellulosic biomass is the most abundant and renewable resource on earth and it is a perfect candidate for the improvement of the current industrial process sustainability since the use of lignocellulosic biomass does not compete with the existing food and feed production^9^.

**Figure 1:**
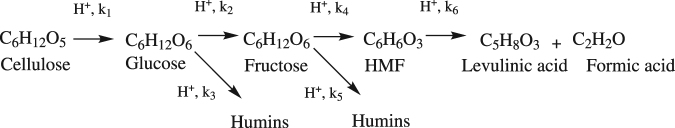
Reaction mechanism for the kinetic study in the conversion of cellulose to HMF.

The conversion of cellulose to HMF is characterized by a series of consecutive reactions (Fig. 1). The pathway starts with a hydrolysis step to depolymerize the cellulosic chain into D-glucose, which is isomerized to D-fructose. After the further dehydration of D-fructose to HMF, HMF can undergo a ring opening reaction in the presence of water. Compared to monomeric sugars, it is more difficult to convert cellulose to HMF, mainly because of two reasons: (i) the hydrolysis of the polymeric chain is required, which takes longer reaction times under harsh reaction conditions, (ii) the hydrolysis step requires the presence of water but this also induces the unwanted ring opening and further conversion of HMF to levulinic acid. These unwanted side reactions can be suppressed with the use of the water immiscible organic solvent methyl isobutyl ketone (MIBK) in the reaction mixture to create a biphasic reaction system. The biphasic reaction system offers the advantage of the *in-situ* extraction of HMF upon formation to the organic phase, hence avoiding the further conversion to levulinic acid or other side products. This results in a higher HMF yield and selectivity. In addition, MIBK is a green solvent and recommended to use by the CHEM21 guide^10^ which ranks solvents based on safety, health and environmental criteria.

The production of HMF occurs in an acidic environment. Generally, two types of acid catalysts can be applied in the conversion process with both their advantages and disadvantages. A first group are the liquid homogenous catalysts (i.e. HCl, H_2_SO_4_, etc.). This type is relatively low-cost and highly effective^11,12^. However, the drawback of homogeneous catalysts is that they are difficult to recover. This leads to excessive amounts of waste streams, which is less appropriate from a sustainable and environmental point of view^12^. A second type of catalysts are the solid heterogeneous acid catalysts (i.e. zeolites, ion exchange resins, etc.). The greatest advantage of this type of catalysts is the ease of separation, and thereby overcoming the recovery difficulties^13^. However, heterogeneous catalysts often suffer from limited activity and selectivity due to lower reaction yields, slower reaction rates and a higher catalyst/substrate ratio compared to homogeneous catalysts^11,12^.

**Figure 2:**
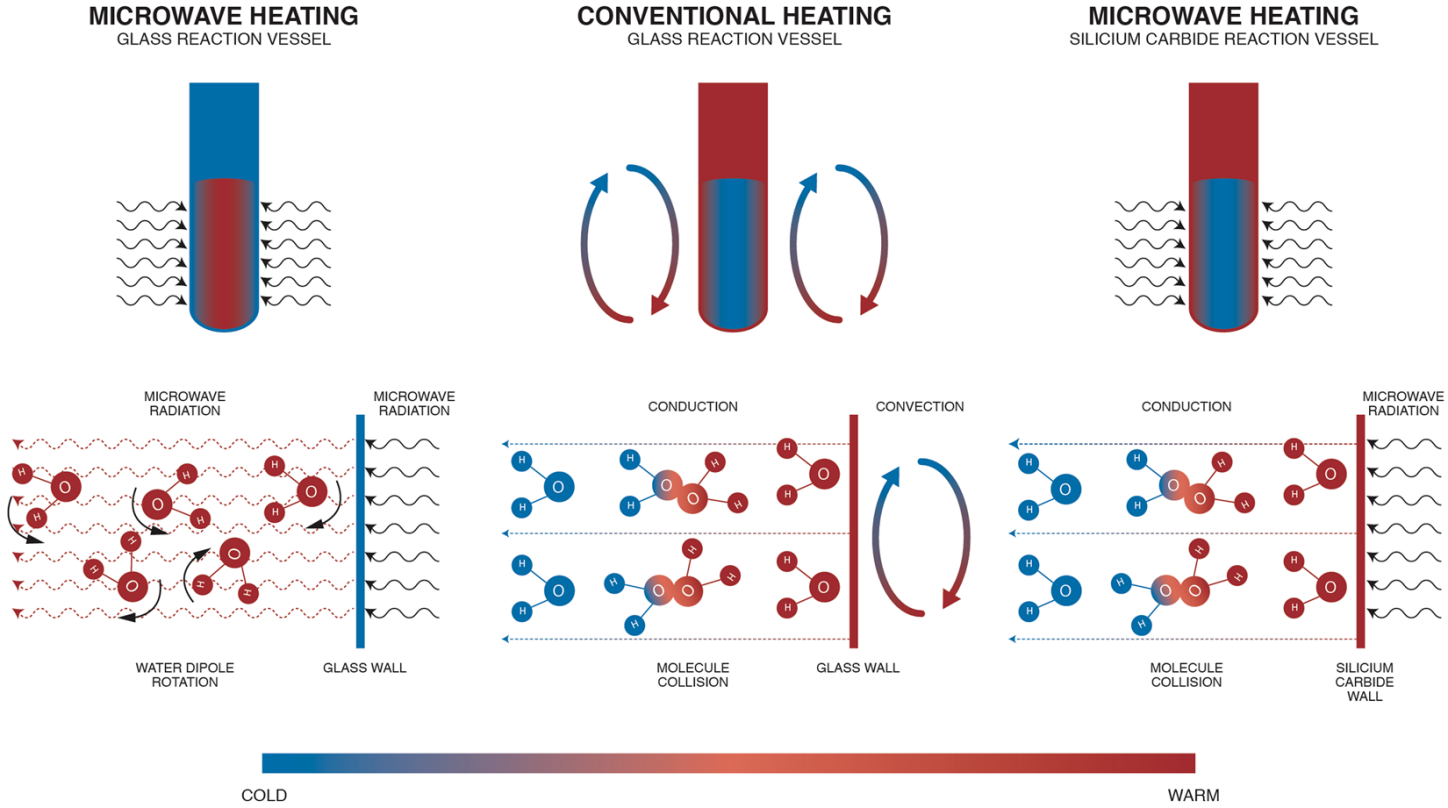
Overview of different heating methods.

The production of HMF requires increased temperatures (>100 °C), which can be supplied by microwave irradiation or conventional heating. An overview of different heating methods, with emphasis on the use of microwaves, is presented in Fig. 2. Microwave irradiation results in an energy-efficient internal heating by direct application of microwave energy on the molecules of the reaction mixture, causing a rapid rise in temperature due to dipole rotation and ionic conduction^14^. Unlike microwave heating, conventional heating comprises a combination of conductive and convective heat transfer which results in (i) a lower heating rate and (ii) the non-uniform heating via the reactor wall (or heat exchanger wall when applicable), leading to local overheating and decomposition of unstable molecules such as HMF^14,15^.

In general, the effect of microwaves can be divided into two categories: (i) thermal effects; where the reaction rate is increased because of the high temperature generated via electromagnetic field and (ii) nonthermal effects; where the interaction with microwave radiation is promoting the bond breaking and bond forming processes in the chemical reaction^16,17^. Nowadays, most of the scientific community agrees that the energy of a microwave photon is far too low to directly cleave molecular bonds and thereby they reject the non-thermal effects of the microwaves^16,18^. However, there are thermal mechanisms of rate acceleration that cannot be achieved or duplicated by conventional heating. These mechanisms are called microwave-specific mechanisms and include the following phenomena: (i) the superheating effect of solvents, (ii) the formation of so-called molecular radiators, (iii) selective heating by using a strongly absorbing microwave catalyst or reagent in a less polar reaction medium and (iv) elimination of wall effects found in convective heating^14,16,17,19^.

In this study, the effect of microwaves was studied using two similar reactors: in one of them, the reaction mixture is heated via direct application of microwave radiation, whereas in the other, conventional wall heating is applied. An identical temperature profile was imposed on both reactors, which enables a proper evaluation of the effect of microwave radiation on the conversion of C6 carbohydrates to HMF. The development of kinetic models were used to generate new insights in the various reaction steps of C6 carbohydrates conversion to HMF.

## Results and Discussion

### Heat transfer assessment

**Figure 3:**
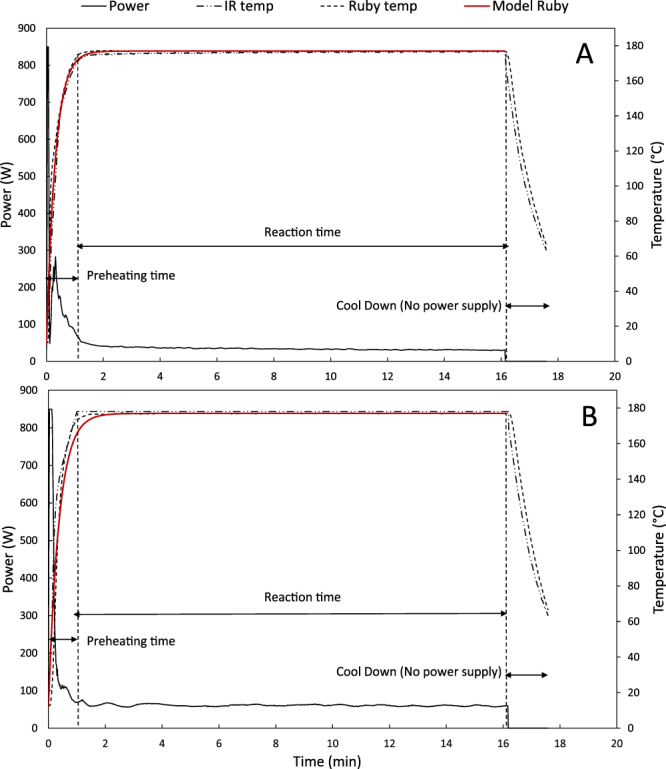
Heating profiles of (**A**) a borosilicate glass reaction vessel and (**B**) a SiC reaction vessel.

In this study, two identically shaped reactors were used: one constructed of borosilicate glass and the other of silicon carbide (SiC). The borosilicate glass reactor allows microwave irradiation to penetrate through the reactor wall into the reaction mixture, hence the heating is caused by dipole rotation and ionic conduction. On the other hand, the SiC reactor shields the reaction mixture from the microwave radiation: SiC is a good microwave susceptor, absorbing all microwave energy readily and strongly^14^. During the experiments, the temperature was monitored as a function of time to assess the various heat transfer steps and the required time to heat up the mixture to the desired temperature (Fig. 3). The results showed that both heating profiles were almost identical and there was no temperature gradient between the reaction mixture (ruby sensor) and the surface of the reaction vessel (IR sensor). The temperature profile as a function of time can be modelled by Equation 1 which fits temperature profiles against the heat balance.

1 $$\frac{d(m{c}_{p}T)}{dt}=UA({T}_{e}-T)$$

In which T represents; the actual temperature of the reaction mixture at the start (°C), t; the time (min), m; the mass of the reaction mixture (kg), c_p_; the heat capacity of the reaction mixture (J kg^−1^ K^−1^), U; the heat transfer coefficient (W m² K^−1^) A; total surface of the reactor (m²); T_e_; the preset reaction temperature (°C).

Assuming that m, c_P_, U and A remain constant during the reaction, these terms can be combined in a constant factor “h” (min^−1^) and Equation 1 can be written as:

2 $$\frac{dT}{dt}=\frac{UA}{m\,{C}_{p}}({T}_{e}-{T}_{i})=h\,({T}_{e}-T)$$

Solving Equation 2 for t = 0 and T = T_i_ (initial temperature of the reaction mixture (°C) results in the following Equation:

3 $$T=h\,({T}_{e}-{T}_{b}){e}^{-ht}$$

The value of h was determined iteratively by fitting the experimental data of the ruby sensor for both reactor types to Equation 3 (using the Excel solver function). The h values were 2.67 min^−1^ and 3.18 min^−1^, for the SiC and borosilicate glass reactor, respectively. These values are very high compared to literature values, confirming that microwave irradiation ensures efficient heating profiles. In a previous study, where stainless steel hydrothermal synthesis reactors were used, an h factor of 0.0095 min^−1^ was determined^20^. Additionally, Girisuta and coworkers reported an h value of 0.359 min^−1^ when using glass ampules with a wall thickness of 1.5 mm^21^.

### Kinetic analysis

The construction and validation of a detailed kinetic model was performed to assess the occurring microwave effects. The reaction mechanism of cellulose to HMF is well described in the literature^1,2,22,23^. In the acidified (HCl) water phase, cellulose is hydrolysed to glucose, and consequently, the glucose is isomerized to fructose. Instantly after the dehydration of fructose to HMF, HMF is extracted to the organic phase. This approach limits the rehydration (ring opening) reaction of HMF to levulinic acid. Patil *et al*. (2012) suggested three parallel reactions form humins from D-glucose, D-fructose and HMF^24^. However, recent studies state that humin formation occurs through condensation polymerization of monosaccharides (i.e. glucose, fructose). Since glucose and fructose are solely soluble in the aqueous phase and HMF is *in-situ* extracted in the organic phase, it is unlikely that HMF is involved in humin formation^25–27^. An overview of the reaction scheme in the presence of an acid catalyst (HCl) is shown in Fig. 1. Based on this scheme, a set of rate equations was developed for each raw material that was used in the experiments, i.e., cellulose, D-glucose and D-fructose. All reaction rate equations are listed in Table 1.

**Table 1:** Overview of rate equations involved in the conversion of cellulose, D-glucose, D-fructose to HMF (with C, G, F, H and L representing cellulose, glucose, fructose, HMF and levulinic acid concentration, respectively, and k_1_, k_2_, k_3_, k_4_, k_5_, k_6_ (h^−1^) are the first order reaction rate constants).

Cellulose feedstock	Glucose feedstock	Fructose feedstock
$\frac{dC}{dt}=-\,{k}_{1}C\,(4)$	$\frac{dG}{dt}=-\,({k}_{2}+{k}_{3})G\,(9)$	$\frac{dF}{dt}=-\,({k}_{4}+{k}_{5})F\,(13)$
$\frac{dG}{dt}={k}_{1}C-({k}_{2}+{k}_{3})G\,(5)$	$\frac{dF}{dt}={k}_{2}G-({k}_{4}+{k}_{5})F\,(10)$	$\frac{dH}{dt}={k}_{4}F-{k}_{6}H\,(14)$
$\frac{dF}{dt}={k}_{2}G-({k}_{4}+{k}_{5})F\,(6)$	$\frac{dH}{dt}={k}_{4}F-{k}_{6}H\,(11)$	$\frac{dL}{dt}={k}_{6}H\,(15)$
$\frac{dH}{dt}={k}_{4}F-{k}_{6}H\,(7)$	$\frac{dL}{dt}={k}_{6}H\,(12)$	
$$\frac{dL}{dt}={k}_{6}H\,(8)$$		

The hence obtained sets of reaction rate equations were solved by using the fourth-order Runge-Kutta method, using the *ode23s* function in Matlab (version R2015a). The kinetic parameters were calculated by fitting the experimental data of each component. The following assumptions were made when developing the kinetic model: (i) the reaction of cellulose to glucose is pseudo-homogeneous irreversible and of first order, all other reactions are irreversible and of first order, and the proton catalyst concentration is considered constant during the reaction so that it can be incorporated into the reaction rate constants, (ii) humins can be formed from glucose, fructose and are soluble in MIBK (since no particulate matter is observed after cellulose conversion).

The experiments were conducted at the following conditions: 177 °C, 0.046 M HCl (pH 1.34) and the feedstock quantity was 25 mg. These conditions were selected because the associated reaction times of up to 4 h enabled to observe the reactions and the effect of microwave radiation (the main aim of this study) over a sufficiently long time to draw valid conclusions. An optimization of the reaction conditions was previously reported in Sweygers *et al*.^22^.

**Figure 4:**
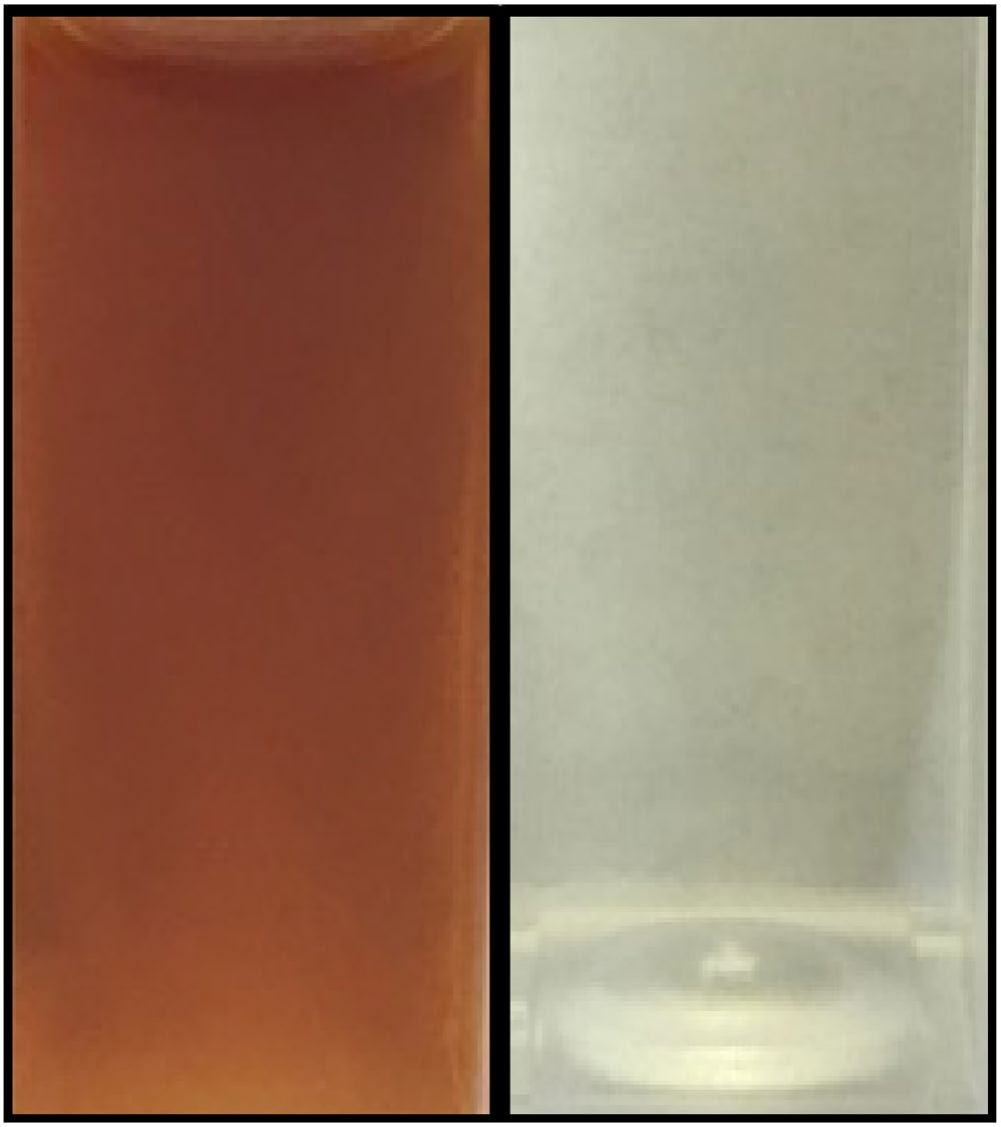
Dark brown-reddish color of the reaction mixture (Left) after a reaction time of 4 h using cellulose and a borosilicate glass reactor containing 1645 ppm HMF and 125 ppm levulinic acid (Right) Transparent color of a standard solution of 2000 ppm HMF and 2000 ppm levulinic acid.

In the proposed scheme in Fig. 1, it is assumed that two parallel reactions cause humin formation from glucose or fructose. Various research papers described the formation of solid particles or so called humins, due to condensation and polymerization reactions of glucose or fructose^25–30^. In a previous study where the MIBK/water reaction system was applied for the optimization of HMF yield, no particulate matter was observed^22^. However, a color shift in the reaction mixture indicated the presence of humins, which are dissolved in the MIBK phase (Fig. 4). The color shift cannot be caused by an increase in HMF concentration, since HMF does not result in a color change when dissolved in MIBK (Fig. 4). Since no analytical methods to quantify dissolved humins are available in the literature, this hypothesis could, however, not be confirmed. In this study, particulate matter was only present in the reaction mixture when using cellulose. Keeping in mind that humins can be soluble in MIBK, all solids can be attributed to the cellulose present in the reaction mixture. Since the concentration of humins was not measured, there will be no further elaboration on the reaction rate constants involving humin formation (k3, k5).

**Figure 5:**
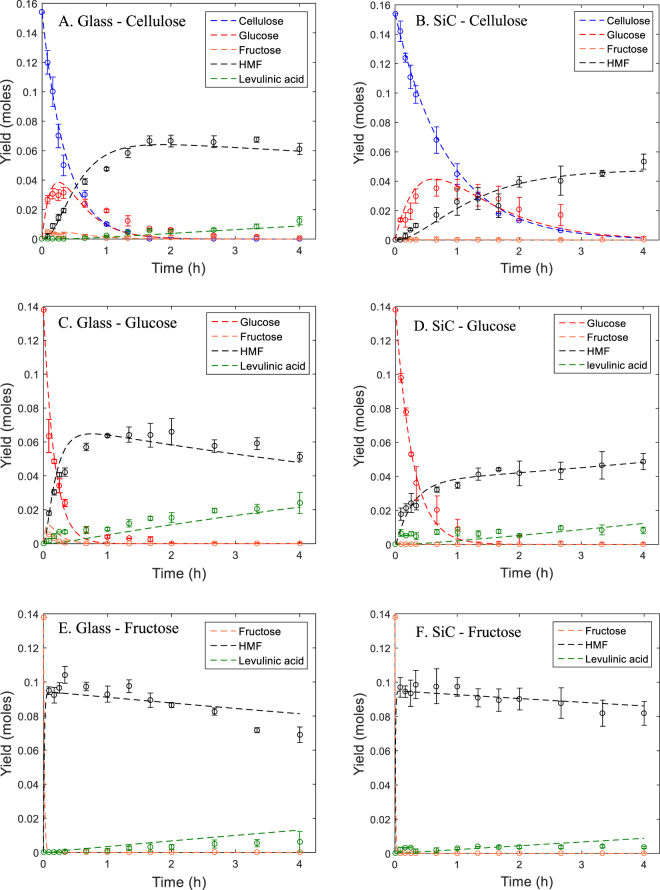
Conversion of cellulose, D-glucose and D-fructose under (Left) microwave irradiation (glass) and (Right) conventional heating (SiC).

The reaction rate constants in Equations 4–15 were calculated by fitting the experimental data of cellulose, glucose, fructose, HMF and levulinic acid (Fig. 5). The calculated values are shown in Table 2. As illustrated in Fig. 5, the experimental results are well predicted by the fitted curves derived from the simulated kinetics of the rate equations. The Runge-Kutta method allows to calculate reaction rate constants of non-measured components. In case of a single reaction with a non-measured component, a correct reaction rate can be obtained based on the mass balance of the reaction. However, in this case the calculated value can differ from the true value of the reaction rate constant, since it is impossible to know the source component (D-glucose, D-fructose) of humin formation.

**Table 2:** Comparison of reaction rate constants between microwave and conventional (SiC) heating calculated by the 4^th^ order Runge-Kutta method. The cells with numbers in bold represent the rate constants involved in humin formation.

Reactor type	Feedstock	Reaction rate constants (h^−1^)					
		k1	k2	k3	k4	k5	k6
Microwave	Cellulose	2.7190	2.4972	**2.9235**	19.4494	**0.6814**	0.0407
	Glucose	—	2.9866	**2.9476**	23.6268	**0.0222**	0.0981
	Fructose	—	–	**—**	50.3815	**23.2135**	0.0375
Conventional	Cellulose	1.1798	0.6484	**1.4266**	115.1500	**0.4627**	—
	Glucose	—	1.1950	**2.3488**	216.4549	**4.6395**	0.0434
	Fructose	—	—	**—**	83.8817	**37.9203**	0.0245

Figure 5 depicts the conversion of cellulose to HMF for the microwave heated system (Fig. 5A) and the conventionally heated system (Fig. 5B). The depolymerization of cellulose occurs more rapidly under microwave heating, thus implying that microwave radiation promotes the depolymerization of cellulose. This observed effect is confirmed by the calculated values of the reaction rate constants k1 for both systems, i.e., 2.7190 h^−1^ and 1.1798 h^−1^ for the glass and SiC vessel, respectively. This means that the depolymerization of cellulose occurs 2.3 times faster under microwave irradiation under the given process conditions (177 °C and 0.046 M HCl). These observed increased reaction rates can be attributed to the softening of cellulose at a temperature of 180 °C, as described in Fan *et al*. (2013). Starting from this temperature, the CH_2_OH groups on the cellulose molecules are subjected to a localized rotation under microwave irradiation, thereby allowing the transfer of microwave energy to the surrounding environment^31^. Because of the limited presence of water inside the cellulose matrix, it is likely that these rotations cause collisions between the CH_2_OH groups and the C1 of a glucose ring, leading to the formation of levoglucosan, which is easily hydrolyzed to glucose. The key temperature of 180 °C is confirmed by several other researchers^32,33^. The effect, where the CH_2_OH groups create microscopic hotspots (acting like a molecular irradiator), can be classified as a specific microwave effect. This specific microwave effect can occur in the isomerization of glucose to fructose since the CH_2_OH group is also present on C1 of glucose. The mechanism where the CH_2_OH group was activated by microwave radiation can be verified by using xylose as a feedstock, which does not contain a CH_2_OH group. A previous study performed by Weingarten *et al*., evaluated the effect of microwave heating on the conversion of xylose to furfural in similar biphasic reaction system (HCl/MIBK), in a dedicated microwave system^34^. This study concluded that microwave heating did not change the kinetics compared to heating by conventional means. This is an indication that the mechanism described in this study and that of Fan *et al*., namely the CH_2_OH group activation, is likely to occur when using C6 carbohydrates as a feedstock^31^. As shown in Fig. 5C,D, the isomerization rate of glucose to fructose is increased by 2.5 fold under microwave irradiation, with a rate constant k2 equal to 2.9866 h^−1^ for the glass vessel and 1.1950 h^−1^ for the SiC vessel. The dehydration of fructose to HMF occurred almost instantaneously in both reactor types, regardless of the feedstock type. This occurrence was confirmed in Fig. 5E,F, where the conversion of all fructose to HMF in five minutes was shown. The dehydration of fructose to HMF is characterized by high rate constants, making it the fastest step in de consecutive reaction of cellulose to HMF. Care needs to be taken when addressing extremely fast reactions, since the compound is consumed so rapidly that it is very difficult to monitor its concentration because the reaction is already finished before an initial reaction rate can be observed. Because of this reason it was impossible to compare the k4 rate constants since no fructose was measured during the reaction. Since HMF can be used as a building block for a variety of fine chemicals (including levulinic acid), a maximal HMF yield is desired. Therefore, in this case the rehydration of HMF to levulinic acid is an unwanted additional step in the consecutive reaction. The biphasic reaction system limits the contact between HMF and water and thereby considerably limits the ring opening reaction to levulinic acid. This is confirmed by the limited rate constants k6: its highest value was only 0.0981 h^−1^ for the conversion of glucose under microwave irradiation. This in contrary to monophasic systems, where the reaction rate of the rehydration of HMF to levulinic acid is characterized as the fastest step in the reaction pathway. In a study performed by Lishi Yan *et al*., a monophasic aqueous reaction system was applied with H_2_SO_4_ as the acid catalyst. The rehydration of HMF to levulinic acid occurred 6 times faster than the hydrolysis of cellulose and the hydration of glucose to HMF^35^. Under conventional heating, even no levulinic acid was detected for the conversion of cellulose (Fig. 5B). Therefore, in this specific case, rate Equation 8 was excluded from the model.

**Figure 6:**
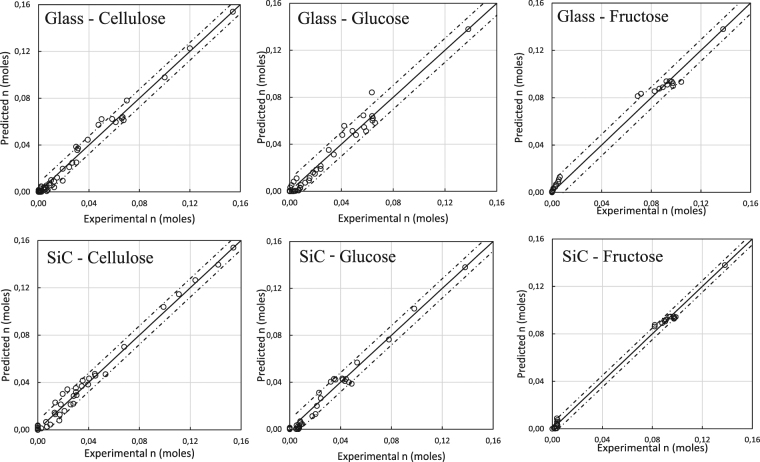
Predicted versus experimental yield (moles). Dashed lines represent the RMSE confidence interval (95%).

The accuracy of the model was validated by plotting the model predicted values against the experimental values. If the proposed model has a good fit, the data points should approach the diagonal of the graph. The confidence interval (CI) is defined by Equations 16 and 17. This statistical procedure was performed for the conversion of each feedstock in both reactor types. Most values are lying inside the 95% RMSE band, implying an overall good model prediction (Fig. 6).

16 $$RMSE=\sqrt{\frac{{\sum }_{j=i}^{n}({P}_{j}-{E}_{i})}{n}}$$

17 $$CI=\overline{x\,}\pm {t}_{95}\ast RMSE$$

RMSE represents the Root Mean Square Error, P_j_; the predicted value of an experiment, E_i_; the experimental value of an experiment, n; number of experiments, $(\bar{x})$; the mean value of an unique experiment, t95; the t value of the inverse two-sided Student-t distribution for a confidence level of 95%.

**Figure 7:**
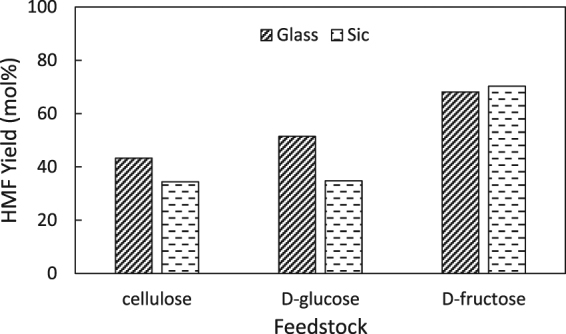
Maximum HMF yield starting from cellulose, D-glucose and D-fructose as a feedstock. The reaction time where maximum HMF is achieved, is depicted above the bar plot. Conditions: T = 177 °C, c_HCl_ = 0.046 M, stirring rate = 800 RPM, reactor content: 25 mg feedstock and 5 mL reaction medium (250 μL acidified water and 4.75 mL MIBK).

The maximum yield of HMF formed from cellulose, D-glucose and D-fructose during microwave and conventional heating is shown in Fig. 7. Under conventional heating, a ranking can be made for the maximum HMF yield: cellulose 43 mol% < D-glucose 51 mol% < D-fructose 68 mol%. The reason for this lower HMF yield from cellulose and D-glucose was related to the reaction pathway (Fig. 1). It is clear that a longer reaction pathway lowers the HMF yields. Adding more steps to the reaction pathway increases the complexity and the presence of unwanted humin side reactions, whilst decreasing the maximal HMF yield. Using conventional heating results in the following ranking by increasing HMF yield: cellulose 34 mol% < D-glucose 35 mol% < D-fructose 70 mol%. The maximum HMF yield was achieved at a reaction time of 240 min when cellulose and D-glucose were used as a feed. When using the Monowave at a reaction temperature of 177 °C, 240 min was the longest reaction time possible. However, the yield of HMF can still increase at longer reaction times and reach a similar yield as under microwave heating (Fig. 5B,D). In the case of D-fructose, the reaction to HMF is completed in 5 min with for both reactor types with an almost identical HMF yield as a result (Fig. 7). If the reaction time could be prolonged when using the silicon carbide reactor with cellulose and D-glucose as a feedstock, a similar HMF would be obtained compared to the HMF yield under microwave radiation. Therefore, it can be concluded that microwaves did not influence the selectivity towards HMF production.

### Microwave energy assessment

**Figure 8:**
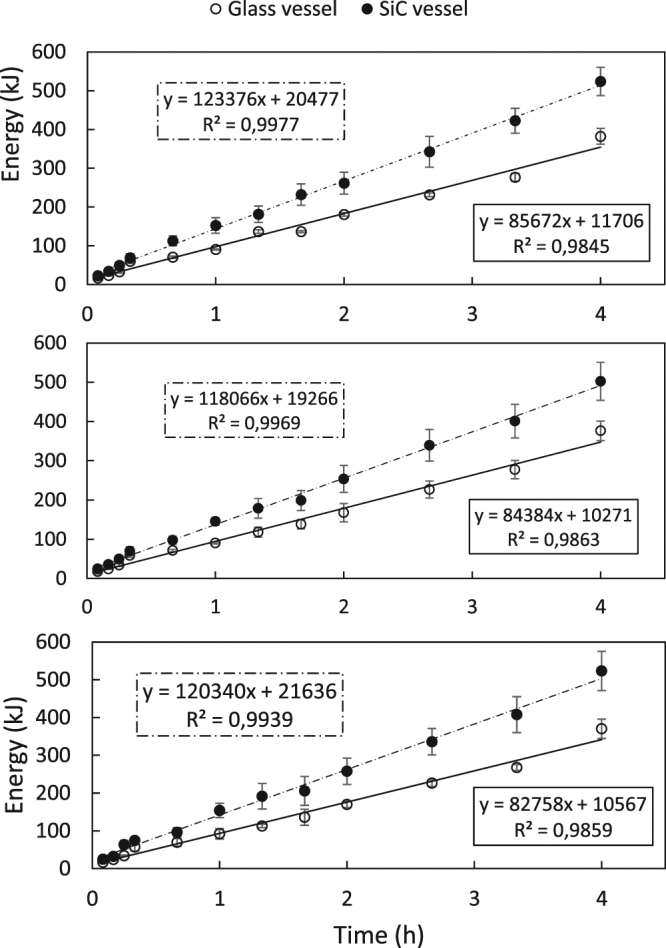
Comparison of energy consumption between a borosilicate glass and SiC reaction vessel using (**A**) cellulose, (**B**) D-glucose and (**C**) D-fructose as a feedstock.

The required microwave energy to heat the reaction mixture and to keep the temperature constant for a specific amount of time was calculated by integrating the power (W) over the reaction time (s). As observed in Fig. 3, most of the power output is required to reach the operating reaction temperature of 177 °C. After the heating stage, the power output remains relatively constant at a lower level to maintain the reaction temperature. The energy profiles for cellulose, fructose and glucose are depicted in Fig. 8. When linear regression is applied, the intercept is the energy required to reach the reaction temperature of 177 °C (since the linear part corresponds to the reaction time). All 3 graphs are showing the same trend, in which conventional heating requires more energy than microwave heating (Fig. 8A–C). This is logical, since there is an extra conduction step under conventional heating, which is necessary for the transfer of microwave energy to the reaction mixture. For example, when using cellulose as a feedstock, 1.75 times (20.477 kJ/11.706 kJ) more energy was required for heating the reaction mixture to 177 °C. Also, the energy requirements for each reactor type were in the same order of magnitude regardless of the used feedstock. When working at long reaction times, the energy required for reaching the reaction temperature was only a fraction of the total energy demand. For instance, a 4 h reaction of cellulose to HMF in a glass reactor required a total energy of 382 kJ, whereas the heating of the reaction mixture only required 20 477 J, being is only 5.3% of the total energy demand. This information indicated that microwave processes could favor the short reaction times. The goal of this research is, however, to evaluate possible microwave effects and an optimization of yield or energy consumption is not in its scope. These findings are in accordance with previous^22^.

## Conclusion

The effect of microwave radiation on the conversion of C6 carbohydrates to HMF in a biphasic reaction system was evaluated by means of a kinetic analysis. The biphasic reaction system assured a high selectivity towards HMF by inhibiting the rehydration of HMF to levulinic acid which was confirmed by very small reaction rate constants (<0.1 h^−1^). The results of this study proved that microwaves have a beneficial effect on the conversion of C6 carbohydrates to HMF. The hydrolysis of cellulose to glucose was increased by a factor 2.3 under microwave irradiation. Also, the isomerization of glucose to fructose shows a similar increase (factor 2.5). The reason behind these results is related to the presence of a CH_2_OH group in glucose and cellulose, which acts as a molecular irradiator. Therefore, the increased reaction rates can be classified as a microwave-specific effect. Applying the microwave energy directly to the reaction mixture led to a highly energy efficient heating method. This was different for the SiC reactor, where a conduction step required up to 1.7 times more energy to reach the reaction temperature of 177 °C. To study the occurrence of microwave effects, long reaction times were applied, which was not favorable in microwave-assisted chemistry. Hence, to make this process more interesting from an economic perspective, a tradeoff needs to be made: moderate conditions (high temperature, low acid concentration) and long reaction times (high energy demand) or more harsh conditions such as high temperature, high acid concentration and short reaction times (low energy demand).

## Materials and Methods

### Chemicals

All experiments were conducted with α-cellulose (Sigma Aldrich), D-glucose (Acros Organics) or D-fructose (Acros Organics). The reactions took place in a biphasic reaction medium consisting of an aqueous phase (demineralized water) acidified with HCl (Fisher Scientific, 37%) and an organic phase consisting of MIBK (Acros Organics, 99.5%).

### Experimental set-up

The experiments were carried out in a dedicated microwave reactor system (Monowave 300, Anton Paar, 2.45 GHz). The temperature was monitored via 2 sensors: a ruby temperature sensor (optic fiber) placed inside the reaction vessel and an IR temperature sensor, which measured the surface temperature of the reactor vessel. The reaction temperature was controlled by the ruby sensor. The volume of the reaction vessel was 10 mL. The reaction vessel was filled with 25 mg of the raw material (cellulose, D-glucose or D-fructose), 250 μL acidified water (0.046 M HCl) and 4.75 mL MIBK. The stirring rate was set to 800 rpm for all the experiments. The maximum power output of the microwave system was set to 800 W. As soon as the reaction time was completed, the reaction vessel was cooled rapidly by placing it in an ice bath to stop the reaction. After cooling, the reaction mixture was filtered (Macherey-Nagel glass fiber filters, GF-3) to remove the particulate matter before analysis. HMF and levulinic acid concentrations were measured in the organic phase. Glucose and fructose concentrations were measured in the aqueous phase. All experiments were conducted in triplicate.

### Analytical techniques

Samples (extract of 1 μL) of the organic phase were analyzed using an Agilent gas chromatograph (Agilent 7890A-series) equipped with a flame ionization detector (FID) to determine HMF and levulinic acid concentrations. The GC was equipped with a HP-FFAP column (Agilent, J&W HP-FFAP length 30 m, internal diameter 0.25 mm, film thickness 0.25 μm). The aqueous phase was analyzed using an Agilent HPLC (Agilent 1100 series) equipped with a HILIC column (Xbridge, HILIC, C18 diameter 2.1 mm, length 150 mm, particle size 3.5 μm) and an ELSD detector (80 °C, 2 L N_2_/min) (Alltech 3300 series). A gradient HPLC method was used for the analysis of glucose and fructose concentration at 70 °C. The mobile phase consisted of solvent A (acetonitrile, Acros Organics) and solvent B (10 mM ammonium formate, Acros Organics) with a flowrate of 0.200 ml/min. The initial mobile phase composition was initially 5% A and was linearly changed to 40% A in 15 min, then kept constant for 5 min, followed by a linear return to the initial conditions in 15 min and kept constant again for 5 min. A micro insert (VWR, 100 μL) was placed inside the HPLC vial, allowing the analysis of small volume samples. The injection volume was 2 μL. The mass of cellulose was gravimetrically determined by filtering the reaction medium through a glass fiber filter (Macherey Nagel, 0.6 μm retention capacity) and drying it for 24 h at 105 °C.

### Data availability

The datasets generated during and/or analysed during the current study are available from the corresponding author on reasonable request.
